# The Timecourse of Electrophysiological Brain–Heart Interaction in DoC Patients

**DOI:** 10.3390/brainsci11060750

**Published:** 2021-06-05

**Authors:** Francesco Riganello, Martina Vatrano, Simone Carozzo, Miriam Russo, Lucia Francesca Lucca, Maria Ursino, Valentina Ruggiero, Antonio Cerasa, Camillo Porcaro

**Affiliations:** 1 S.Anna Institute—Research in Advanced Neurorehabilitation, 88900 Crotone, Italy; m.vatrano@isakr.it (M.V.); simone.carozzo@gmail.com (S.C.); russo.miriam@libero.it (M.R.); l.lucca@isakr.it (L.F.L.); mariaursino@alice.it (M.U.); valentinaruggiero.1@gmail.com (V.R.); antonio.cerasa@irib.cnr.it (A.C.); camillo.porcaro@istc.cnr.it (C.P.); 2 Institute for Biomedical Research and Innovation (IRIB)—National Research Council of Italy (CNR), 87050 Mangone, Italy; 3 Department of Information Engineering, Università Politecnica delle Marche, 60131 Ancona, Italy; 4 Centre for Human Brain Health, School of Psychology, University of Birmingham, Birmingham B15 2TT, UK; 5 Institute of Cognitive Sciences and Technologies (ISTC) - National Research Council (CNR), 00185 Rome, Italy

**Keywords:** disorders of consciousness, EEG, heart rate variability, circadian rhythms, behavioral response

## Abstract

Disorders of Consciousness (DOC) are a spectrum of pathologies affecting one’s ability to interact with the external world. Two possible conditions of patients with DOC are Unresponsive Wakefulness Syndrome/Vegetative State (UWS/VS) and Minimally Conscious State (MCS). Analysis of spontaneous EEG activity and the Heart Rate Variability (HRV) are effective techniques in exploring and evaluating patients with DOC. This study aims to observe fluctuations in EEG and HRV parameters in the morning/afternoon resting-state recording. The study enrolled 13 voluntary Healthy Control (HC) subjects and 12 DOC patients (7 MCS, 5 UWS/VS). EEG and EKG were recorded. PSDalpha, PSDtheta powerband, alpha-blocking, alpha/theta of the EEG, Complexity Index (CI) and SDNN of EKG were analyzed. Higher values of PSDalpha, alpha-blocking, alpha/theta and CI values and lower values of PSD theta characterized HC individuals in the morning with respect to DOC patients. In the afternoon, we detected a significant difference between groups in the CI, PSDalpha, PSDtheta, alpha/theta and SDNN, with lower PSDtheta value for HC. CRS-R scores showed a strong correlation with recorded parameters mainly during evaluations in the morning. Our finding put in evidence the importance of the assessment, as the stimulation of DOC patients in research for behavioural response, in the morning.

## 1. Introduction

Disorders of Consciousness (DOC) are a spectrum of pathologies affecting one’s ability to interact with the external world. It can be either due to a traumatic cause [1,2], non-traumatic cause [3,4], or a combination of both [5] and gives rise to ethically challenging questions [6,7,8], including the end-of-life decisions.

For clinical purposes, consciousness is commonly defined by wakefulness (i.e., the presence of spontaneous periods of opening the eyes) and awareness (i.e., the ability for a subject to respond to the internal/external stimuli in an integrated way). Two possible conditions of patients with DOC are Unresponsive Wakefulness Syndrome/Vegetative State (UWS/VS) [9] and Minimally Conscious State (MCS) [10]. The first is characterized by a spontaneous opening of the eyes and no sign of consciousness but reflexive responses to external stimuli [11,12]; the second condition exhibits minimal but discernible signs of non-reflex behaviours which occur reproducibly (yet inconsistently) as a response to visual, auditory, tactile, or noxious stimuli.

The DOC cover a broad population of very heterogeneous pathologies with diverse etiologies, injuries, and outcomes, making it hard to distinguish in the clinical practice between the different levels of consciousness [6], leading the examiners to a possible misdiagnosis [13,14,15,16]. The clinical assessment is based on clinical consensus and behavioural scales, such as the Coma Recovery Scale-Revised (CRS-R) that represent the current “gold standard” for diagnosing patients with DOC [17,18]. The difficulty in assessing the consciousness level of UWS patients often depends on a subjective interpretation of the observed spontaneous and volitional behaviour [19]. In the absence of speech or cognitive functions, the motor response is the only way to observe evidence of consciousness in these patients [20,21,22]. A critical challenge in clinical practice is minimizing the diagnostic error to make a correct prognosis, the appropriate treatments, and facilitate end-of-life decisions [23,24]. Therefore, DOC patients’ behavioural fluctuation can significantly impact diagnostic accuracy [25].

Analysis of spontaneous EEG activity and the Heart Rate Variability (HRV) are effective techniques in exploring and evaluating patients with DOC [26,27]. Studies have evidenced a relationship between cortical EEG rhythms and general cognition in resting-state condition [28,29]. Decrement of posterior alpha rhythms was observed in subjects with mild cognitive impairment compared with normal elderly subjects [30,31]. The summation of post-synaptic potentials at their apical dendrites reflects the temporal synchronization of cortical pyramidal neurons in the EEG activity [32]. The EEG theta (range of frequency 4–7 Hz) and alpha band (range of frequency 8–12 Hz) constitute a significant neural substrate for human cognition [33,34,35,36]. The ratio between EEG alpha-band power recorded with open and closed eyes represents the reduction in spontaneously-recorded occipital band power at open eyes, and its circuits are thought to be involved in the consciousness [37]. The alpha-theta (α/θ) ratio was found to discriminate Alzheimer’s disease from healthy older controls, patients with mild and severe Alzheimer’s disease discriminate individuals with and without cognitive impairment in older individuals with Parkinson’s disease [38,39,40].

The Central Autonomic Network (CAN) is an integrative model that describes the interaction between neural structure and heart function involved and functionally linked in the affective, cognitive and autonomic regulation [41,42,43]. The principal neural structure of the CAN covers the brainstem (periaqueductal grey matter, nucleus ambiguous, and ventromedial medulla), limbic structures (amygdala and hypothalamus), prefrontal cortex (anterior cingulate, insula, orbitofrontal, and ventromedial cortex) and cerebellum [41,44]. HRV reflects the complex interaction between the brain and the cardiovascular system and describes the Autonomic Nervous System (ANS) functional setup [45,46,47]; evidence suggests these measures also reflect (to some extent and indirectly) higher brain functions and are reliable, independent indicators of Central Nervous System (CNS)/ANS interaction [48,49]. The HRV is generally analyzed in the time and frequency domains [46]. However, the physiological phenomena that characterize the biological events are dynamic and complex [50]. Because of the dynamic and complex nature of the physiological phenomena that characterize the biological events, the non-linear analysis represents a valuable approach to understanding brain–heart two-way interaction [43].

In the time domain, the standard deviation of EKG Interbeat interval (IBI) calculates with the ectopic beat removed (SDNN) represents the sympathovagal system’s contribution to the HRV. In the non-linear domain, the HRV entropy quantifies the interbeat interval’s unpredictability and complexity (IBI) series. Higher and lower entropy indicate, respectively, higher or lower unpredictable IBI sequence, and correspondingly a higher or lower Heart–Brain two-way interaction [51]. Approximate entropy (ApEn) and sample entropy (SampEn) allow quantifying time series data’s complexity like heart rate intervals [52].

Sample Entropy (SampEn) [53] and Multiscale Entropy [54] are among the most commonly applied non-linear analysis methods used in the HRV analysis. SampEn is a modification of ApEn and has been suggested to be independent of the data length. Given a series of data length N, defined the embedding dimension (m) and the tolerance (r), SampEn (N, m, r) is the negative value of the logarithm of the conditional probability that two similar sequences of m points remain similar at the next point m + 1 counting each vector over all the other vectors except on itself [53].

MSE investigates the information content in non-linear signals at different temporal scales (coarse-graining), generally using the SampEn to quantify the degree of unpredictability of time series [54].

The Complexity Index (CI) is calculated from the MSE measures and is the sum of the entropies computed for different scales (i.e., at different levels of resolution of the signal). Thus the CI provides a scalar score, which is the aggregation of MSE over multiple time scales, allowing to get insights into the integrated complexity of the brain–heart interaction.

However, it is well known that behavioural responses are influenced by circadian rhythms, with the suprachiasmatic nuclei (central pacemaker) influences adaptation and behavioural response to the environment [55,56,57]. Alteration of sleep/wake cycle could influence the fluctuation during the day in arousal and response to the command that potentially can confound the clinical assessment reliability in DOC patients [19,58,59]. Studies evaluating daily electrophysiological imbalances between heart–brain activities in DOC patients are lacking. For this reason, this study aims to observe fluctuations in EEG and HRV parameters in the morning/afternoon resting-state conditions. With this aim, we analyzed the power-spectrum density in alpha (PSDα) and theta (PSDθ), αB, α/θ, CI and SDNN. In particular, we expect to find (1) higher EEG PSDα, αB, α/θ, SDNN and CI in HC when compared to the DOC patients; (2) higher PSDα, SDNN and CI and lower PSDθ in the morning compared to the afternoon.

## 2. Materials and Methods

We enrolled 13 voluntary Healthy Control (HC) subjects (5 male, age 47 ± 7, 8 female, age 43 ± 5) and 12 patients with DOC, 7 diagnosed as MCS (3 male, age 55 ± 14, 4 female, age 60 ± 17) and 5 as UWS/VS (3 male, age 61 ± 18, 2 female, age 48 ± 21) (Table 1). The consciousness diagnosis was based on clinical consensus and CRS-R assessment [5]. The enrolled patients were hospitalized in a special rehabilitation unit for patients with severe DOC at the S. Anna Institute of Crotone (Italy). The inclusion criteria were: (1) age more than 16, (2) no administration of neuromuscular blockers or sedation within 24 h of enrolment, (3) eyes opening (indicating wakefulness and rest cycles), (4) diagnosis of UWS or MCS, based on behavioural assessments by way of CRS-R [17], (5) stable clinical condition. Exclusion criteria were: (1) documented history of brain injury; (2) previous psychiatric or neurologic disorders; (3) administration of pharmacological drugs interacting with the level of consciousness.

This study was carried out following the rules of the Declaration of Helsinki of 1975 (https://www.wma.net/what-we-do/medical-ethics/declaration-of-helsinki/, 20 January 2021) revised in 2013.

All participants were recorded by EEG and EKG in two time windows: during the morning (between 08:00 and 09:00) and afternoon (between 14:00 and 15:00). The patients were assessed by CRS-R in the morning. To avoid differences in the EEG/EKG recording, due to a different rehabilitative programme, the patients underwent the rehabilitation activity plan after the first EEG/EKG recording in the morning until noon, and no rehabilitative activity was planned before the second EEG/EKG recording.

The EEG/EKG recording was done in the electrophysiology laboratory in the absence of transient noise, with natural light equipped with a Faraday cage. EEG/EKG record lasted 5 min, 5 with closed eyes. Five minutes of EEG with open eyes were also recorded to calculate the αB. For the DOC patients, the closed eyes condition was maintained by a bandage.

### 2.1. Data Recording

EEG and EKG signals plus the EOG for the eye blink were acquired at 1024 Hz by the Neurowave device (http://khymeia.com/prodotti/neurowave/ 20 January 2021). A 32 channel cap with sintered Ag-AgCl ring electrodes was used for the EEG recording, and two polygraphy channels for the EKG (recorded by adhesive electrodes positioned on the chest) end the EOG.

Recording impedances were kept <5 kΩ. All signals were recorded with common reference; the signal ground was connected to an electrode on the left arm. Signals were filtered between 0.1 and 200 Hz and stored on a hard disk for offline processing.

### 2.2. Data Extraction

EEG signals were extracted and analyzed by EEGLAB (https://sccn.ucsd.edu/eeglab/index.php, 20 January 2021) [60] and visually controlled for artefacts. After artefacts remotion by Independent Component Analysis (ICA) method, alpha and theta PSD were extracted.

EKG was analyzed by Kubios advanced software for HRV analysis [61]. The signals were controlled for artefacts and ectopic beats removed by the interpolation method. For the EKG analysis, the CI and the SDNN conditions were calculated.

The CI was based on the MSE approach quantifying the degree of irregularity over a range of coarse-grained scales (τ) from 1 to 5. The coarse-grained were constructed by averaging the IBI/tachogram’s data points within non-overlapping windows of increasing length τ. For each coarse-grained scales, the Sample Entropy was calculated, and the CI extracted as the sum of the Sample Entropy for each coarse-grained scale. The parameters m and *r* of SampEn were set to 2 and 0.2, respectively.

### 2.3. Data Analysis

For the statistical analysis was used the non-parametric exact test. This approach provides more accurate results when the sample size is small or in the case of tables sparse or imbalanced [62].

A Mann–Whitney exact test compared HC and Patients for PSD α and θ, αB, α/θ, SDNN and CI in the morning and the afternoon. Morning vs. afternoon was compared for each group by Wilcoxon exact test for EEG α and θ PSD, αB, α/θ, SDNN and CI.

The effect size was calculated as the absolute value of *Z*/√(N), where *Z* is the *Z*-statistic [63,64] of the statistical test and N is the total number of subjects. The effect size results were considered: *r* < 0.1 not significant; 0.1 ≤ *r* < 0.3 low; 0.3 ≤ *r* < 0.5 medium; *r* ≥ 0.5 high. Correlation between recorded parameters and CRS-R was observed by Spearman’s test. The level of significance was set at *p* ≤ 0.01.

## 3. Results

**Figure 1:**
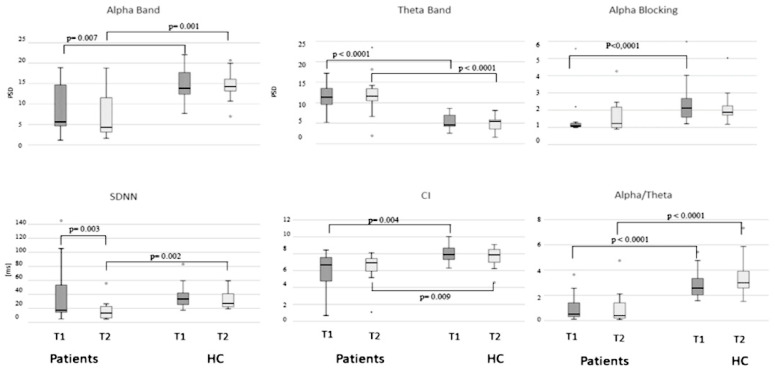
Boxplot of patients and healthy control (HC) compared for PSDα, PSDθ and alpha-blocking are displayed in the first line and SDNN, whereas Complexity Index (CI) and ratio alpha/theta in the second line. Morning recording in dark grey (T1) and afternoon recording in light grey (T2).

In the morning, we detected significant differences in the comparison between DOC and HC for PSDα, PSDθ, α/θ, αBand CI metrics (Mann–Whitney exact test: −3.699 ≤ *Z* ≤ −2.488, 0.0001 ≤ *p* ≤ 0.007, 0.37 ≤ *r* ≤ 0.52). Otherwise, In the afternoon significant additional differences were detected for PSDα, PSDθ, α/θ, CI, and SDNN (Mann–Whitney exact test: −3.481 ≤ *Z* ≤ −2.339, 0.0001 ≤ *p* ≤ 0.009, 0.33 ≤ *r* ≤ 0.49) (Figure 1).

**Figure 2:**
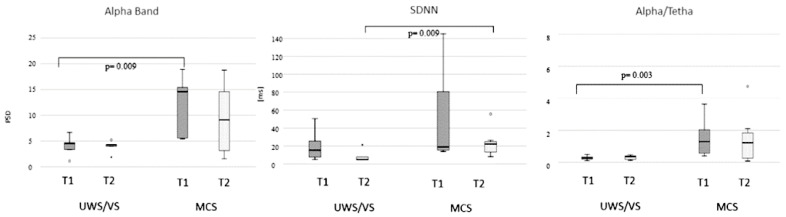
Boxplot of unresponsive wakefulness syndrome/vegetative state (UWS/VS) and minimally conscious state (MCS) patients compared for PSDα, SDNN and alpha/theta. Morning recording in dark grey (T1) and afternoon recording in light grey (T2).

Comparing MCS and UWS, significant differences were in the morning for PSDα (Mann–Whitney exact test: *Z* = −2.355, *p* = 0.009, *r* = 0.48) and α/θ, (Mann–Whitney exact test: *Z* = −2.680, *p* = 0.003, *r* = 0.59), and in the afternoon for SDNN (Mann–Whitney exact test: *Z* = −2.355, *p* = 0.009, *r* = 0.48)(Figure 2).

Comparing morning vs. afternoon, only in DOC patients showed a significant difference for the SDNN (Wilcoxon exact test: *Z* = −2.589, *p* = 0.003, *r* = 0.53) (Figure 1).

**Figure 3:**
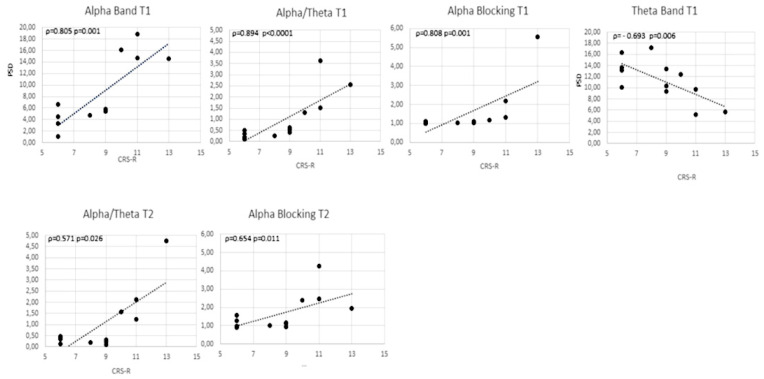
Scatter plot of the correlation between Coma Recovery Scale-Revised (CRS-R) and PSDα, ratio alpha/theta, alpha-blocking and PSDθ in the morning (first line), and between CRS-R and ratio alpha/theta and alpha-blocking in the afternoon (second line).

The CRS-R was positively correlated at Spearman correlation’s test, with PSDα (*ρ* = 0.805, *p* = 0.001), α/θ (*ρ* = −0.894, *p* = 0.0001), αB in the morning (*ρ* = 0.809, *p* = 0.001), and negatively with PSDθ (*ρ* = −0.693, *p* = 0.006). In the afternoon a positive correlation was for α/θ (*ρ* = −0.571, *p* = 0.026), and αB (*ρ* = 0.654, *p* = 0.011) (Figure 3).

## 4. Discussion

Our study confirms that DOC patients are characterized by evident electrophysiological heart–brain fluctuations along the day with an evident reduction in the early afternoon [19,58]. This change in the level of consciousness due to the fluctuation of the arousal makes significantly challenging the interpretation of inconsistent behaviours or possible simple motor response [65].

Recording the resting-state EEG in the morning and the afternoon allows us to detect a significant difference between HC and DOC patients in several metrics, such as CI, PSDα, PSD θ and α/θ. In particular, we found higher values of PSDα and α/θ in HC and higher PSDθ for DOC patients. Moreover, additional significant differences were found for αB only in the morning and the SDNN only in the afternoon, with higher values for the HC. Similar results were detected comparing MCS and UWS/VS, with a significant difference in the morning for PSDα and α/θ, and in the afternoon for the SDNN. Finally, comparing the recorded parameters morning vs. afternoon, lower values of SDNN were found for the DOC patients in the afternoon. These findings are in line with those observed in previous studies. Indeed, higher values in the EEG theta power spectrum were documented in UWS/VS patients than MCS and healthy subjects [66,67,68]. Alpha and Theta EEG components were also observed associated with consciousness, with higher theta and lower alpha PSD detected in UWS/VS patients [30,67,69]. Finally, reduced alpha activity accompanied with higher power theta components was associated with worse clinical outcomes [27].

Interestingly we found that significant differences between HC and DOC patients were for αB only in the morning and the SDNN only in the afternoon, with higher values for HC. Danze and colleagues [70] reported that in around 10% of VS patients, the EEG is nearly normal late in the course of the disease but without evidence of αB. Classically, αB is the reduction in spontaneously-recorded occipital alpha band power in response to the opening of the eyes [71] and was associated with visual attention [72,73]. Differently from the cortical alpha that is thought to be generated by feedforward and feedback interactions between the thalamus and overlying cortex [74,75], blocking is considered to arise from changes in the phase synchrony of populations of these near-identical cortico-thalamic alpha oscillators [32,76] and is thought to be involved in consciousness circuitry [37]. Otherwise, the CI expresses the complex brain–heart two-way interaction. This dynamic process shows a reduced or absent complexity in pathological conditions [47,51]. The loss of significance observed in the differences between HC and DOC for αB in the early afternoon is compatible with reducing arousal in HC, which, however, shows a higher cardiac variability (SDNN).

Comparing MCS and UWS/VS, significant differences were found in the morning for PSDα and α/θ, with MCS patients showing the highest alpha power activity. However, these differences decreased in the afternoon. Similar to the HC, the difference between MCS and UWS/VS was for the SDNN in the afternoon, with higher values for MCS. We also found that the alpha/theta ratio was higher in HC than DOC and higher in MCS than UWS/VS. This evidence is in agreement with Lechinger et al. [67], although this difference was not evident between MCS and UWS/VS in the afternoon. Similarly, the correlation between CRS-R and αB and alpha/theta ratio were higher in the morning than in the afternoon. The fluctuation of arousal and consequently of the consciousness level could be related to the circadian rhythms of EEG alpha and theta band [77], of the Central/Autonomic Nervous System [57], abnormal sleep/wake cycle [59,78]. Nevertheless, these fluctuations might also be related to disturbed sleep/wake cycles due to modifications of the environment surrounding the patient caused by procedures of the staff and/or some interaction modalities of the relatives that may affect residual endogenous mechanisms of self-regulation [79]. The correct alternation between periods of sleep and wakefulness deriving from intact circadian rhythms’ system seems central for adequate arousal levels and then for consciousness [80]. It is crucial to consider that in UWS/VS patients, if it is possible to observe the presence of alternate periods of wakefulness and sleep, this does not imply preserved circadian sleep/wake cycling [81]. On the nocturnal melatonin levels and light-induced melatonin suppression, a study report altered levels of melatonin secretion and reduced light-induced melatonin suppression in UWS/VS patients [82]. Blume and colleagues find that higher levels of integrity in melatonin sulfate and temperature circadian rhythms are related to a better behavioural repertoire [83]. This finding implies that alteration in these circadian rhythms may also interfere in the observed fluctuation in EEG and EKG observed parameters.

A limitation to our study is due to the small sample size and age heterogeneity between patients and HC. If the used statistical approach considers these issues, the limited number of patients might cause low accuracy in results, as reported for the effect size [84]. For these reasons, our results need to be confirmed in a greater cohort of DOC patients.

## 5. Conclusions

For the first time, our study provides a daily electrophysiological timecourse of the functional imbalance characterizing the heart–brain relationship in the DOC patients. Despite the small sample size, the combined EEG and HRV analysis confirm that physiological parameters are nearer to normal values in the morning than in the afternoon and that the incomplete recovery of the circadian rhythms as the sleep/wake cycle might influence the reliable assessment of patients with severe DOC. Our study highlights the importance of considering the variability in behavioural response in DOC patients, which could induce misdiagnosis. Indeed, as already reported by our group in previous studies [19,58], the morning could be ideal for observing response in DOC patients. Again, these findings highlight how in DOC patients, the fluctuation in the arousal and alteration of the circadian rhythms (as observed in and the abnormal sleep/wake cycle) might influence the behavioural response, as also observed in the electrophysiological recordings.

## Figures and Tables

**Table 1:** Demographics information.

SUBJECT	GENDER	AGE	FROM EVENT TO HOSPITALIZATION (DAYS)	ETIOLOGY	DIAGNOSIS	CRS
**1**	M	72	40	HEM	MCS	11
**2**	F	71	26	HEM	MCS	9
**3**	F	72	36	HEM	MCS	9
**4**	M	71	22	TBI	UWS	6
**5**	F	65	14	HEM	MCS	9
**6**	F	33	35	TBI	UWS	6
**7**	M	40	39	HEM	UWS	8
**8**	F	63	53	ISC	UWS	6
**9**	M	73	49	ISC	UWS	6
**10**	M	45	26	ISC	MCS	13
**11**	F	34	34	HEM	MCS	10
**12**	M	48	34	HEM	MCS	11
**13**	M	48			HC	
**14**	M	40			HC	
**15**	F	36			HC	
**16**	F	38			HC	
**17**	M	51			HC	
**18**	F	40			HC	
**19**	F	51			HC	
**20**	F	45			HC	
**21**	M	40			HC	
**22**	M	55			HC	
**23**	F	49			HC	
**24**	F	40			HC	
**25**	F	46			HC	

M: Male; F: Female; HEM: Hemorrhage; TBI: Traumatic Brain Injury; ISC: Ischemic; MCS: Minimally Conscious State; UWS/VS: Unresponsive Wakefulness Syndrome; HC: Healthy Controls.

## Data Availability

The data presented in this study are available on request from the corresponding author.
